# DIY 3D Microparticle Generation from Next Generation Optofluidic Fabrication

**DOI:** 10.1002/advs.201800252

**Published:** 2018-06-01

**Authors:** Kevin S. Paulsen, Yanxiang Deng, Aram J. Chung

**Affiliations:** ^1^ Department of Mechanical, Aerospace, and Nuclear Engineering Rensselaer Polytechnic Institute (RPI) Troy NY 12180 USA; ^2^ Engineering Directorate Lawrence Livermore National Laboratory (LLNL) Livermore CA 94550 USA; ^3^ School of Biomedical Engineering Korea University Seoul 02841 Republic of Korea

**Keywords:** 3D microparticles, inertial microfluidics, optofluidic fabrication, optofluidics

## Abstract

Complex‐shaped microparticles can enhance applications in drug delivery, tissue engineering, and structural materials, although techniques to fabricate these particles remain limited. A microfluidics‐based process called optofluidic fabrication that utilizes inertial flows and ultraviolet polymerization has shown great potential for creating highly 3D‐shaped particles in a high‐throughput manner, but the particle dimensions are mainly at the millimeter scale. Here, a next generation optofluidic fabrication process is presented that utilizes on‐the‐fly fabricated multiscale fluidic channels producing customized sub‐100 µm 3D‐shaped microparticles. This flexible design scheme offers a user‐friendly platform for rapid prototyping of new 3D particle shapes, providing greater potential for creating impactful engineered microparticles.

Engineered microparticles with nonspherical shapes have attracted increased attention recently due to different shape‐dependent functionalities that emerge from specific particle geometries.^1^ Examples of these shape‐dependent functionalities are seen in biology where the shape and surface area of drug carrier particles can alter cellular uptake,^2^ and the shape of implanted hydroxyapatite particles can change the body's inflammatory response.^3^ Additionally, complex double‐cone particles have allowed for programmable release of multiple active ingredients for therapeutic applications.^4^ Besides for bioinspired applications, nonspherical particles can self‐align in a confined flow^5^ or change the rheological properties of a solution.^6^ Also, particles with interlocking shapes can create low‐density loadbearing structures from particle jamming.^7^ Although, it remains challenging to create complex 3D‐shaped particles to meet new emerging demands.

Microfluidic methods such as stop flow lithography (SFL)^8^ and optofluidic maskless lithography (OFML)^9^ have shown great potential for producing complex‐shaped particles, however, the approach mostly resulted in 2D extrusion shapes with uniform side profiles. By modifying the SFL process, we previously demonstrated the creation of 3D‐shaped particles with a fabrication scheme called optofluidic fabrication.^10^ Optofluidic fabrication creates an infinite set of complex 3D‐shaped particles simply by varying inertial flow^11^ and light conditions. However, our 3D particles generated from the use of this method have been mainly on the mm scale because of large mm scale channels used to lower pressure drops, limiting the method's potential usage. Similar particle fabrication methods termed optical transient liquid molding (TLM)^12^ and Dean flow‐based optofluidic fabrication^13^ were reported generating 3D‐shaped particles with overall sizes of hundreds of micrometers. These methods created sub‐millimeter particles by using smaller channel molds, even though their dimensions are still large for potential applicability in numerous biotechnological applications.^14^ Here, we present a novel method termed next generation optofluidic fabrication (NG‐OF) for creating 3D‐shaped microparticles an order of magnitude smaller than our previously demonstrated particles, while still utilizing primarily mm scale channels for ease of fabrication and low pressure drops.

As shown in **Figure** **1**a, NG‐OF uses multiscale channels where the inertial flow shaping is achieved in a mm scale channel upstream,^10^ and passes a tapered reduction section while keeping its flow shape. The flow is then rapidly stopped, ultraviolet (UV) polymerization is initiated in the reduction section (Figure 1b; and Figure S1, Supporting Information), and a 3D microparticle(s) is generated (Figure 1c–e). Furthermore, we present a new rapid channel fabrication scheme to effectively create fluidic channels with custom pillar configurations. When a new particle shape is needed, a new channel with a different pillar configuration is required. Thus, instead of preparing new channels either by photolithography or 3D printing,^10^, ^15^ NG‐OF uses an on‐the‐fly approach that creates pillars at a desired channel location by local UV polymerization (see Figure 4a and more details below) that is used for creating 3D‐shaped microparticles.

**Figure 1:**
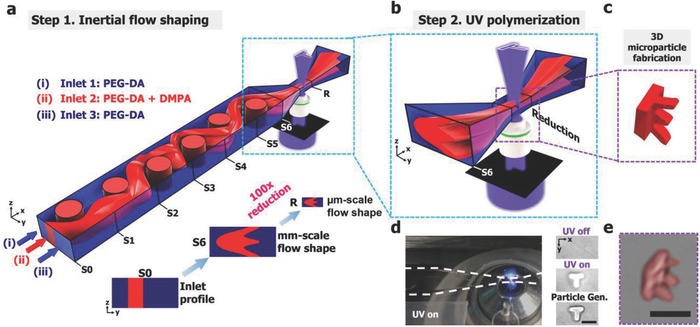
Operating principles of next generation optofluidic fabrication: a) The flow cross‐section of inert (i and iii) and UV‐reactive streams (ii) are shaped by fluid inertia as they flow past pillars at *Re* ≈ 5. The flow cross‐sections at slices S0 and S6 are shown at the inlet and after passing six pillars. After inertial flow shaping, fluid streams enter a tapered reduction zone that reduces the channel area 100‐fold, scaling down the flow cross‐section to the µm scale. b) After inertial flow shaping, the flow is quickly stopped, and patterned UV light is illuminated on the channel reduction section creating a 3D microparticle. c) A rendering of a microparticle resulting from a T‐shaped UV light pattern with an E‐shaped flow cross‐section. d) Experimental setup showing the microparticle polymerization process. From left to right: Far‐field view of UV illumination on the channel reduction; From top to bottom: Before, during, and after 20 ms UV illumination. e) 3D view of an experimentally fabricated microparticle. Scale bar represents 75 µm.

As mentioned, NG‐OF is based on inertial flow shaping from our previous optofluidic fabrication.^10^ Figure 1a shows inertial flow shaping of three streams in a fluidic channel containing six pillars. The initial and final flow shapes are shown at corresponding slices labeled S0 and S6. The channel design is comprised of mm and µm scale channels connected through a tapering section. This design is adopted to avoid excessively high input pressures when small microchannels are used for inertial flow (Note S1, Supporting Information), which is often not compatible with conventional Polydimethylsiloxane (PDMS)‐glass fluidic devices. With our design, in upstream mm scale channels, inertial flow shaping can occur under reasonable pressure drops while maintaining the flow cross‐section through the tapering zone. The flow is then rapidly stopped, and a 3D particle is generated by illuminating patterned UV light at the reduction section (labeled “R”), as shown in Figure 1b–e.

To generate a well‐defined particle, the flow needs to be stopped fully to allow sufficient UV exposure and minimize diffusion between UV‐curable and inert fluids. The stopping time relies on two timescales: 1) hydraulic capacitance and 2) viscous dissipation.^12^ First, the hydraulic capacitance timescale is dominated by the flexible channel walls which inhibits fast changes in pressures due to expansion and relaxation of the PDMS channel walls under pressure.^8^, ^16^ When the input pressure is lowered to stop the flow, the channel relaxes and induces unwanted squeeze flow.^8^, ^16^ We address this issue by using stiffer thermally cured PDMS channels, however, more curing agent or other rigid oxygen permeable materials^17^ can further reduce this hydraulic capacitance timescale. Second, the viscous dissipation timescale is determined by viscous diffusion of momentum, proportional to $D_{h}^{2}$/ν, where ν is the kinematic viscosity. This implies that smaller channels will result in faster viscous dissipation leading to shorter flow stopping time. However, the higher pressures required by smaller channels often become too high for conventional PDMS‐glass channels to withstand. As a solution, NG‐OF utilizes the tapered reduction design where the channel cross‐sectional area decreases by a factor of 100. This allows a moderate input pressure of 1200 mbar to still generate sufficient inertial secondary flows at *Re* = 5 (where *Re* is the Reynolds number and see Note S1 (Supporting Information) for detailed definitions of all associated parameters) with stop times less than 400 ms (see Figure S2, Supporting Information).

For NG‐OF microparticle generation, we first prepared a channel mold using a 3D printer (photocurable inkjet printer) since photolithography is not practical for creating multiscale channels with a vertical tapering design. To understand and characterize the inertial flow in our channel, entire channels were simulated using COMSOL Multiphysics. Note that the 3D printed mold displayed a rounded cross‐sectional channel shape in the reduction section due to resolution limits of the 3D printer (Figure S3, Supporting Information), and this feature was taken into consideration for simulations. Simulation details can be found elsewhere,^15^ but briefly, the steady‐state incompressible Navier–Stokes and convection‐diffusion equations were solved simultaneously with each model containing 8.9 × 10^6^ degrees of freedom. Normalized concentration slices c* of photoinitiator concentration are presented in **Figure** **2**a–c where three channels with different pillar configurations are modeled: A channel with six pairs of half‐cylindrical pillars located along the side‐walls (Figure 2a), six pillars located along the center (Figure 2b), and pillars that create an “E”‐shape flow cross‐section (Figure 2c). Channel top views are also presented for an *x*–*y* slice 5 µm above the channel bottom, and more slices can be found in Figure S4 (Supporting Information). The *z*–*y* slices S0‐S6 show the flow shape evolution, and the final reduction slice labeled “R” is shown with the area scaled up 100 times for easier visual inspection.

**Figure 2:**
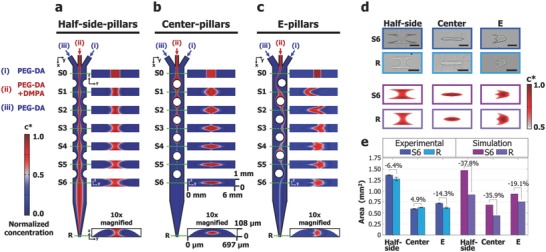
Numerical versus experimental analysis of inertial flow shaping with reduction channels. a) Normalized concentration plots of inertial flow shaping from a channel with half‐cylindrical pillars located along the side walls. The *x*–*y* view shows the concentration 5 µm above the channel bottom. *z*–*y* slices at S0‐S6 show the flow shaping achieved upstream. After the reduction section, slice R shows the miniaturized flow cross‐section with the area scaled up 100 times for easier visual inspection. b) Similar concentration plots are shown for a channel with pillars located along the central axis and c) for a complex pillar configuration that results in an “E” shape flow profile. d) Thin particles are polymerized and flipped on their sides at locations S6 and R to reveal flow shape changes. Scale bars for row S6 represent 1 mm, while scale bars for row R represent 100 µm. Simulation cross sections at similar locations as d) are shown using an upper 50% concentration threshold. e) To compare cross‐sectional shapes pre‐ and postreduction, the reduction cross‐sections were scaled up. Error bars represent standard deviation from 15 particles created in 3 different channels.

These simulation concentration slices were compared with experiments. The simulation concentration slices were thresholded similar to our previous work^15^ using the upper local 50% concentration range to compare with the experimental particle shapes. For experiments, a thin slit of UV light was illuminated at locations S6 and R across the entire width of the channel, generating a particle whose shape represents the flow cross‐section. Simulation and experimental cross‐sections for the three channel designs are shown in Figure 2d, and more particle images can be seen in Figure S5 (Supporting Information). Channel heights were normalized between S6 and R (Figure S6, Supporting Information) to allow direct comparison between cross‐sectional area measurements, and a detailed comparison is plotted in Figure 2e. Although general cross‐sectional area trends matched, slight cross‐sectional area differences were seen. These cross‐sectional area differences were smaller for experimental cases compared to simulations cases. Since the steady state simulation results do not take into account the flow stopping step, diffusion occurring during flow stoppage creates a larger experimental particle shape, especially in the channel reduction where particle sizes approach relevant diffusion scales. One interesting feature was seen in the center‐pillar case (Figure 2b) where the experimental area was increased while the simulation predicted a decrease. This may be because the numerical prediction does not include particle polymerization or oxygen inhibition. For the center‐pillar channel, the flow shape is isolated from the side walls, preventing loss of polymerization from oxygen‐inhibition near the side walls,^18^ allowing the full uninhibited flow shape to polymerize. Also, it was assumed that the main channel was perfectly rectangular. Under internal pressure, PDMS channels bulge, leading to shape discrepancies which could be corrected with more accurate geometry modeling.

With the channel design validated as a method to miniaturize the inertially shaped flow, the same three channels were used to create 3D‐shaped microparticles as shown in **Figure** **3**. The full 3D particle shape could be adjusted by simply changing the light pattern or changing the location the light pattern was illuminated. In Figure 3a, UV light was illuminated off‐center, and 3D microparticles with overall dimensions of width × depth × height approximately equal to 70 × 70 × 104 µm^3^ are generated. By using the center‐pillar channel in Figure 3b, the UV‐reactive stream is directed away from the channel walls, allowing for a smaller 3D particle. Although the flow cross section is not as intricate from this pillar configuration, overall particle dimensions are as small as 30 × 50 × 35 µm^3^. To demonstrate increased particle shape complexity, the E‐channel was used (Figure 3c) that created particles with dimensions of ≈70 × 70 × 104 µm^3^. Note that since the photomasks were aligned manually, some shape discrepancies can be seen from the particle side‐views. However, these mask alignment discrepancies could be fixed completely with a digital micromirror device (DMD) UV system to accurately control the location of UV illumination.[[qv: 9b]] These 3D microparticles can potentially be made smaller and finer by overcoming challenges with diffusion between coflowing fluid streams. As particle sizes become smaller, diffusion length scales can approach particle sizes, either requiring faster flow stopping to limit diffusion or particles with less intricate shapes. With the experimentally tested conditions of *Re* = 5, the average transit time through the main channel section is 2.2 s, leading to a total diffusion time of ≈2.6 s, including flow stopping. The theoretical particle resolution can be inferred by calculating the diffusion length *L*_D_ from Equation (1) below

(1) $$L_{D}=\sqrt{D_{\mathrm{PI}}t_{D}}$$

where *D*_PI_ is the diffusivity of photoinitiator and *t*_D_ is the total diffusion time. With the DMPA diffusivity of 4 × 10^−11^ m^2^ s^−1^ 15 in poly(ethylene glycol) diacrylate (PEG‐DA) and a diffusion time of 2.6 s, an isometric diffusion length of ≈10 µm is expected, representing the particle resolution limit. By incorporating a rigid channel,^19^ higher allowable pressures and flow rates could reduce transit and stop times, leading to finer particle features. For example, a Reynolds number of 20 reduces the transit time fourfold to 0.55 s. Assuming a faster stop time of <100 ms from a rigid channel, the diffusion length could be reduced to *L*_D_ ≈ 5 µm, allowing for particle features half the size.

**Figure 3:**
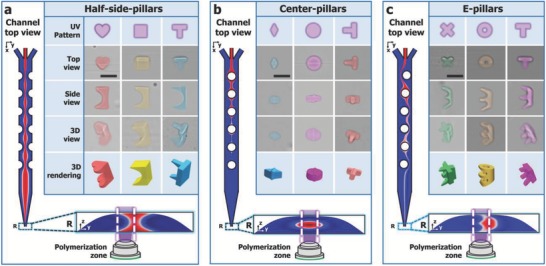
Examples of 3D‐shaped microparticles created using NG‐OF. a) The top‐view, side‐view, and 3D‐view of experimentally fabricated 3D microparticles are shown by combining three different UV light patterns with part of the “I”‐shaped flow cross‐section. b) The center‐pillar design creates a smaller UV‐reactive flow shape. c) The E‐pillar channel demonstrates more complex 3D‐shaped microparticles. Renderings of the 3D particles are shown below. The position of UV illumination is also illustrated with the UV light path intersecting the channel cross section. Particles are false‐colored to enhance contrast with background. Scale bars represent 75 µm.

It should be mentioned that to fabricate a new particle shape, a specific pillar configuration should be determined. For this task, two open‐source programs, Flowsculpt^20^ and uFlow,^21^ can be used to easily design a new channel. Flowsculpt calculates the necessary pillar configuration to achieve a desired flow shape, and uFlow quickly displays the flow shape from user inputted pillars. Although these tools are not designed specifically for the NG‐OF process due to the interpillar distance difference, they still provide quick and very close approximations to flow shapes and channel designs that greatly aid in the design process. Then, a specific channel can be prepared using a 3D printer; however, the 3D printing preparation process can be costly and time‐consuming whenever new channels are required. Therefore, instead of printing new channels, a simple method is hereby presented by creating custom pillars in blank channels via UV polymerization. As shown in **Figure** **4**a–c, a blank channel is first filled with UV‐reactive fluid. Then a circular pattern of UV light is illuminated to create a pillar (Note S2, Supporting Information) at a desired location. By using a programmable motorized microscope stage (Movie S1 and Figure S7, Supporting Information), multiple pillars were created sequentially with high precision in a fully automated manner. We refer to this custom UV‐polymerization pillar scheme as “Do‐it‐yourself” (DIY) pillars. Although a blank channel mold originally needs to be printed, any pillar configuration (size, location, and spacing) can be prepared from this single blank channel mold.

**Figure 4:**
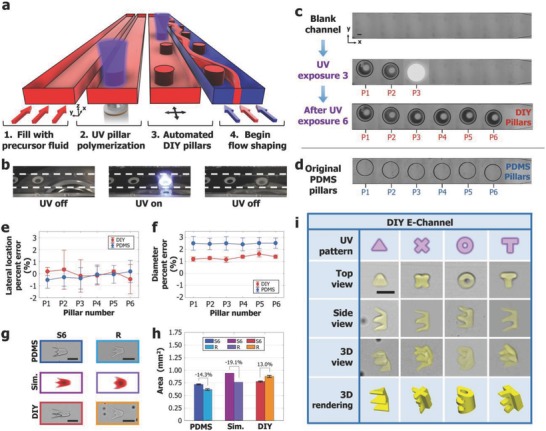
Overview of on‐the‐fly pillar fabrication for microparticle generation. a) A schematic representation of the on‐the‐fly pillar fabrication process used to create custom DIY pillars from UV‐polymerization. 1) An empty channel is filled with UV‐reactive fluid. 2) A circular photomask is illuminated to polymerize a custom pillar. 3) A motorized stage moves to the next pillar location, and this process is repeated until all pillars are created. 4) Then streams of UV reactive precursor and inert fluids are injected for to initiate flow shaping. b) Far field image showing a DIY pillar being polymerized. c) Experimental images showing the DIY pillar polymerization process to replicate d) the E‐channel with PDMS pillars from the 3D printed channel mold. e) The lateral pillar locations and f) the diameter of DIY and original PDMS pillars are compared to the intended channel design for the E‐channel. Error bars represent the standard deviation from 3 separate channels. g) Thin particles are polymerized and flipped on their sides at locations S6 and R to reveal flow shape changes. Scale bars for column S6 represent 1 mm, while scale bars for column R represent 100 µm. The reduction cross‐sections were scaled up to set the channel heights equal. h) Cross‐sectional areas are plotted for the original channel, simulation thresholded results, and DIY E‐particles. Error bars represent standard deviation from 15 particles created in 3 different channels. i) 3D‐shaped microparticles created using a DIY Channel. Scale bar represents 75 µm.

Note that under normal UV polymerization conditions, the DIY pillars will not remain fixed during flow due to the oxygen permeable layer. Thus, we overexpose UV light and use a slight channel modification made during the channel preparation step. An oxygen diffusion barrier is added on top of the channels by simply inserting a conventional glass slide into the liquid PDMS during the curing process. This glass slide becomes embedded within the PDMS channel structure and controls oxygen diffusion into the channel. Using the DIY‐pillar generation scheme, we created the same channel layout as the E‐channel used in Figures 2 and 3 (Movie S1, Supporting Information). The lateral location and diameter of the six sequential pillars were analyzed (Figure S8, Supporting Information) and plotted in Figure 4e,f. Both the original 3D printed channel molds and the DIY channels were compared. For both the original channels and the DIY channels, the lateral positions of the pillars and errors in diameter were compared (Figure 4e,f), and resulted in less than 3% error, showing a high precision pillar fabrication scheme.

Simulation and experimental areas were analyzed (Figure 4g) at S6 and R for the DIY channel and compared with the E‐channel data from Figure 2d. The E‐shape is stretching relatively more during inertial flow shaping and also shows slight asymmetry in the middle “tip.” This stretched inertial flow shaping may be caused from the more rigid DIY pillar channels since the embedded glass diffusion barrier does not allow as much channel expansion during flow. The different E‐particle shapes might also be caused from slight concave shape (Figure S9, Supporting Information) of DIY pillars due to the numerical aperture (NA) of the 2.5× objective (NA = 0.085). The experimental cross‐sectional areas were plotted for the DIY case and shown in bar plots in Figure 4h next to the original channel and simulation area results. Unlike the PDMS and simulation area changes, the DIY‐E particle area increases 13.0% after the taper. However, the DIY E‐shapes are actually matching more closely to the simulations compared to the original E particles, possibly since the DIY pillar diameters are closer to the intended computer‐aided design (CAD) than the original PDMS pillars (Figure 4f). 3D microparticles were also created using the DIY E‐channels with four different photomasks, shown in Figure 4i. Similar to the original channels, these 3D particles had dimensions of ≈70 × 70 × 104 µm^3^.

In summary, the presented next generation optofluidic fabrication provides a simple platform to create 3D‐shaped microparticles by overcoming many design challenges faced when scaling down channel sizes with inertial flows. By utilizing primarily mm scale fluidic channels, low operating pressures allow for fast flow response times while still using standard PDMS channels. NG‐OF can rapidly create DIY channels with any pillar configuration from a single blank channel mold. The versatility of NG‐OF allows for biological components such as cells or drugs to be added to precursor streams and become encapsulated within microparticles for studies of cell‐particle interactions with complex‐shaped particles. 3D particles from NG‐OF can also benefit from the increased surface area per unit volume to be used for highly sensitive RNA/DNA sensing over 2D microparticles.^22^ Moreover, other additives such as magnetic nanoparticles can be added for particle control using an external magnetic field.^12^ The presented work uses 3D printed molds to create multiscale channels. By using 3D printers with finer resolution, or by using a process such as micromachining, smaller reduction channels could be created that easily allow smaller 3D microparticles. With the ability for NG‐OF to quickly create a variety of 3D‐shaped microparticles, this technique can be easily adopted to meet the demands of new emerging fields.

## Conflict of Interest

The authors declare no conflict of interest.

## Supporting information

Supplementary

Supplementary
