# Supplementary material for: DIY 3D Microparticle Generation from Next Generation Optofluidic Fabrication
Source: Adv Sci (Weinh). 2018 Jun 1;5(7):1800252. doi: 10.1002/advs.201800252 (PMC6051230; doi:10.1002/advs.201800252)
Supplement: Supplementary file 1 — Supplementary [file ADVS-5-1800252-s001.pdf]

Copyright WILEY-VCH Verlag GmbH & Co. KGaA, 69469 Weinheim, Germany, 2018.

ADVANCED SCIENCE
Open Access

Supporting Information

for *Adv. Sci.,* DOI: 10.1002/advs.201800252

DIY 3D Microparticle Generation from Next Generation Optofluidic Fabrication

*Kevin S. Paulsen, Yanxiang Deng, and Aram J. Chung**

Supporting Information

DIY 3D Microparticle Generation from Next Generation Optofluidic Fabrication

*Kevin S. Paulsen, Yanxiang Deng, Aram J. Chung**

Dr. K. S. Paulsen, Y. Deng, Prof. A. J. Chung
Department of Mechanical, Aerospace, and Nuclear Engineering
Rensselaer Polytechnic Institute (RPI)
Troy, NY 12180, USA
E-mail: chunga6@rpi.edu

Dr. K. S. Paulsen
Engineering Directorate
Lawrence Livermore National Laboratory (LLNL)
Livermore, CA 94550, USA

Prof. A. J. Chung
School of Biomedical Engineering
Korea University
Seoul 02841, Republic of Korea
Email: ac467@korea.ac.kr

[Note S1]

Pressure drop analysis: When scaling down a channel, higher hydraulic resistance increases the pressure drop across the channel necessary to match the Reynolds number. The necessary pressure increase can be calculated using a hydraulic circuit model and solving for the pressure drop ΔP across a channel as a function of the Reynolds number. The pressure drop in a channel can be calculated as follows:

$$\Delta P = \mathrm{Q} R_h \# (\mathrm{S1})$$

where Q is the volumetric flow rate and R_h is the hydraulic resistance. The hydraulic resistance for a square channel can be approximated using Equation S2[1],

$$R_h = 28.4 \frac{\mu L}{D_h^4} \# (\mathrm{S2})$$

where μ is the dynamic viscosity, L is the channel length, and D_h is the hydraulic diameter. The Reynolds number can then be solved in terms of the volumetric flow rate, as follows,

$$Re = \frac{\rho U_{\mathrm{avg}} D_h}{\mu} = \frac{\rho (\mathrm{Q}/D_h^2) D_h}{\mu} = \frac{\rho \mathrm{Q}}{\mu D_h} \# (\mathrm{S3})$$

where ρ is the density and U_{avg} is the average flow velocity. Finally, by solving Equation S3 for Q and plugging into Equation S1 in combination with Equation S2 allows the estimation of the pressure drop as follows,

$$\Delta P = 28.4 \frac{Re \mu^2 L}{D_h^3} \# (\mathrm{S4})$$

Equation S4 implies that when matching the Reynolds number in a channel that is 10 times smaller, the pressure drop across the channel increases by a factor of 10^3.

[Note S2]

UV illumination conditions and throughput: UV light is patterned using a photomask, demagnified through an objective, and focused on the fluidic channel (**Figure 1**b, d). The necessary UV exposure time for polymerization depends on UV power, channel height, and objective. For example, a 2.5× objective is useful for polymerizing particles at the millimeter scale owing to a large field-of-view. However, when creating microparticles, the UV pattern can be demagnified to a smaller field-of-view, thus increasing the UV power density. Therefore, higher magnification objectives can polymerize smaller particles with shorter UV exposure times, although limited field-of-views and higher numerical apertures make higher magnified objectives less suitable for large and tall channels. Because of the larger S6 channel dimensions (6 mm × 1 mm), a 2.5× objective with a large field-of-view was used for polymerizing the S6 particles with 2 s UV exposure. For polymerizing the particles in the reduction section at location R, a 20× objective was used with 20 ms UV exposure due to the higher UV power delivery achieved from greater demagnification in the smaller channel section (~600 µm × 100 µm). After polymerizing a particle, the NG-OF process can be repeated with a cycle time of approximately 10 s. Particles were created with a throughput of 360 particles/hour in the presented work. Using photomasks with multiple patterns placed side-by-side, at least 10 particles could be polymerized at a time based on the current 1,200 µm wide field-of-view, thus leading to a potential throughput of 3,600 particles per hour.

To create a DIY pillar and overcome the oxygen inhibition layer, a 10× objective was used with a higher UV power density of >200 mW cm^{-2} to create an “anchor” pillar (see Movie S1). Subsequently, a 2.5× objective with a UV power density of ~30 mW cm^{-2} created the final pillar around the anchor pillar. This two-step process ensures that the anchor pillar is fixed in

place using the more powerful UV exposure, while the less powerful 2.5× objective could still create the large millimeter scale pillars from the larger field-of-view. Longer UV exposures were observed to create larger pillar diameters (**Figure S10**). However, little growth was observed after 8 s of UV exposure, and thus a UV exposure of 8 s was chosen in this study.

[Note S3]

Experimental Section

Channel Fabrication: Polydimethylsiloxane (PDMS) channels from Sylgard 184 Silicone Elastomer (Dow Corning Corporation, MI, USA) were created using soft lithography on 3D printed channel molds. An Objet500 3D printer was used with the VeroWhitePlus material (Purple Porcupine, CA, USA) with a specified manufacturer x–y resolution of 42 µm, and a 16 µm layer thickness for printing channel molds. PDMS was prepared using a base-to-curing agent ratio of 5:1 to help stiffen the channels[2] for faster flow stop times (approximately 15% decrease in flow stoppage at *Re* = 5). The uncured PDMS was poured over channel molds, desiccated to remove air bubbles, and was allowed to cure at room temperatures for 24 hours to prevent heat warping of the 3D printed channel molds. For blank DIY channels, a glass slide was inserted into the liquid PDMS during curing to embed the glass within the channel. After curing, a biopsy punch (Ted Pella, CA, USA) was used to punch inlet and outlet holes in the unbonded PDMS channel. The unbonded channel, as well as a 127 mm PDMS coated glass slide were then treated with air plasma (Harrick Plasma, NY, USA) for 20 s at 800 mTorr. The PDMS channel was then bonded to the slide and further cured in an oven at 90°C for 24 hours to stiffen the PDMS.

Precursor Fluids and Flow Control: Inert fluid streams (sheath fluid) were made of poly(ethylene glycol) diacrylate M_n 250 (Sigma–Aldrich, MO, USA), while UV-reactive fluid streams consisted of PEG–DA with 5 wt% 2,2-dimethoxy-2-phenylacetophenone (DMPA) (Sigma–Aldrich, MO, USA). To help visualize flow, 15 µm polystyrene beads were added to all fluid streams (Thermo Fisher Scientific, MA, USA). Precursor fluids were contained in glass media bottles with ported caps (Kinesis, Inc, IL, USA) for inlet tubing and air pressure control. A programmable pressure control system was used to control the inlet pressure of fluid streams.

The height of the outlet waste container was adjusted to prevent excessive hydrostatic pressure induced flow. It was observed that with input pressures greater than 2000 mbar, channel delamination could occur owing to the PDMS bonding integrity. Note that this issue can be potentially resolved by creating PDMS molds printed using a higher resolution 3D printer that could allow stronger PDMS bonding and higher operating pressures.

Imaging and UV Illumination: A Phantom v7.3 high-speed camera (Vision Research, NJ, USA) was used to image all experiments which were performed on a Zeiss Axio Observer A1 (Carl Zeiss, Germany) inverted microscope. A SCAN Märzhäuser motorized stage (Carl Zeiss) was used with a custom LabVIEW VI to control the x–y stage position and automate the DIY pillar creation process. A Lumen 220 UV light source (Prior Scientific, MA, USA) was used to direct 365 nm UV light through the microscope objectives and onto the channels. A Lambda SC mechanical shutter (Sutter Instrument, CA, USA) was used to control the exposure of UV light. A photomask (CAD/Art Inc., OR, USA) was placed in the microscope field stop to create patterned UV light.

[Figure S1]

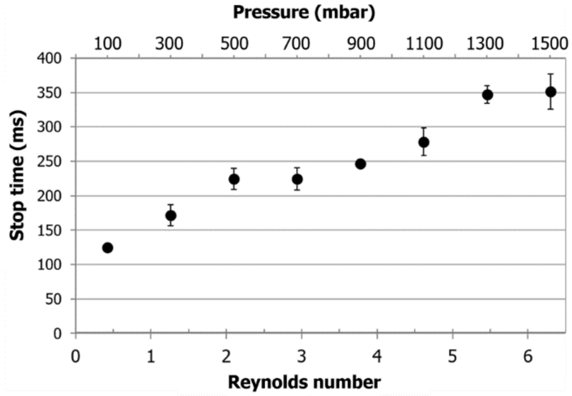

Figure S1. Flow stop time characterization. Flow stop times *vs.* Reynolds numbers (and pressures) for a center-pillar channel when fully loaded media bottles were used. Error bars represent the standard deviations of the stop times from three different trials.

[Figure S2]

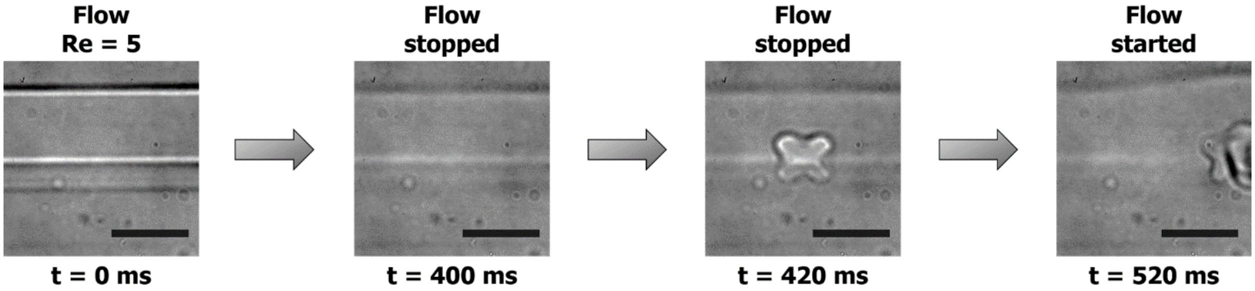

Figure S2. Microparticle fabrication. After the inertial flow shaping, the flow is stopped in 400 ms, patterned UV light is illuminated for 20 ms, and a 3D microparticle is flushed away as the process repeats. The scale bar represents 75 µm.

[Figure S3]

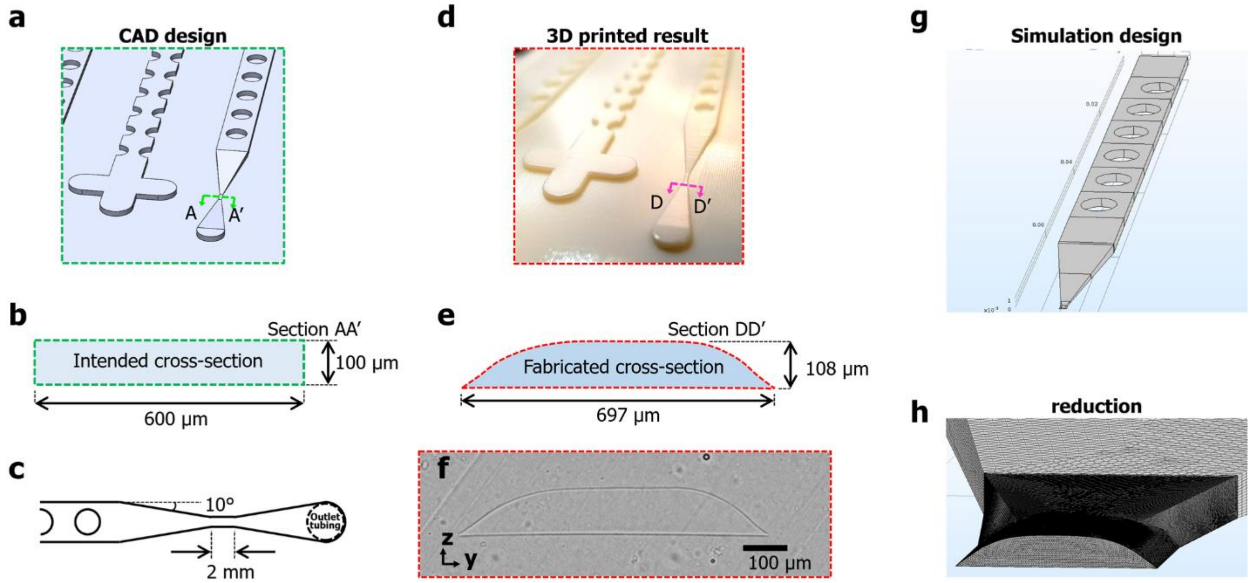

Figure S3. **Channel geometries based on designed and fabricated results**. **a)** The tapered reduction section is designed to reduce the channel cross-section from 6 × 1 mm^2 to 600 × 100 μm^2. **b)** The designed reduction cross-section shown at section AA' is rectangular and maintains the same 6:1 aspect ratio. **c)** A top view schematic shows the x–y taper angle of 10° leading to a 2 mm long reduction section. **d)** 3D printed channel mold with the reduction section labeled as DD'. **e)** The 3D printed channel mold has a rounded cross-section illustrated based on the section DD' owing to the intrinsic resolution limits of the photocurable inkjet 3D printer. **f)** Experimental cross-section of the reduction channel. A PDMS channel created from the 3D printed mold was filled with photocurable PEG–DA, and a thin slit of UV light was illuminated to polymerize a thin particle. The cross-section of the displayed particle represents the cross-sectional shape of the channel mold. **g)** The simulated channel design was rectangular except for the reduction **h)** where the cross-sectional shape transitions towards the actual rounded cross-section from the 3D printed mold.

[Figure S4]

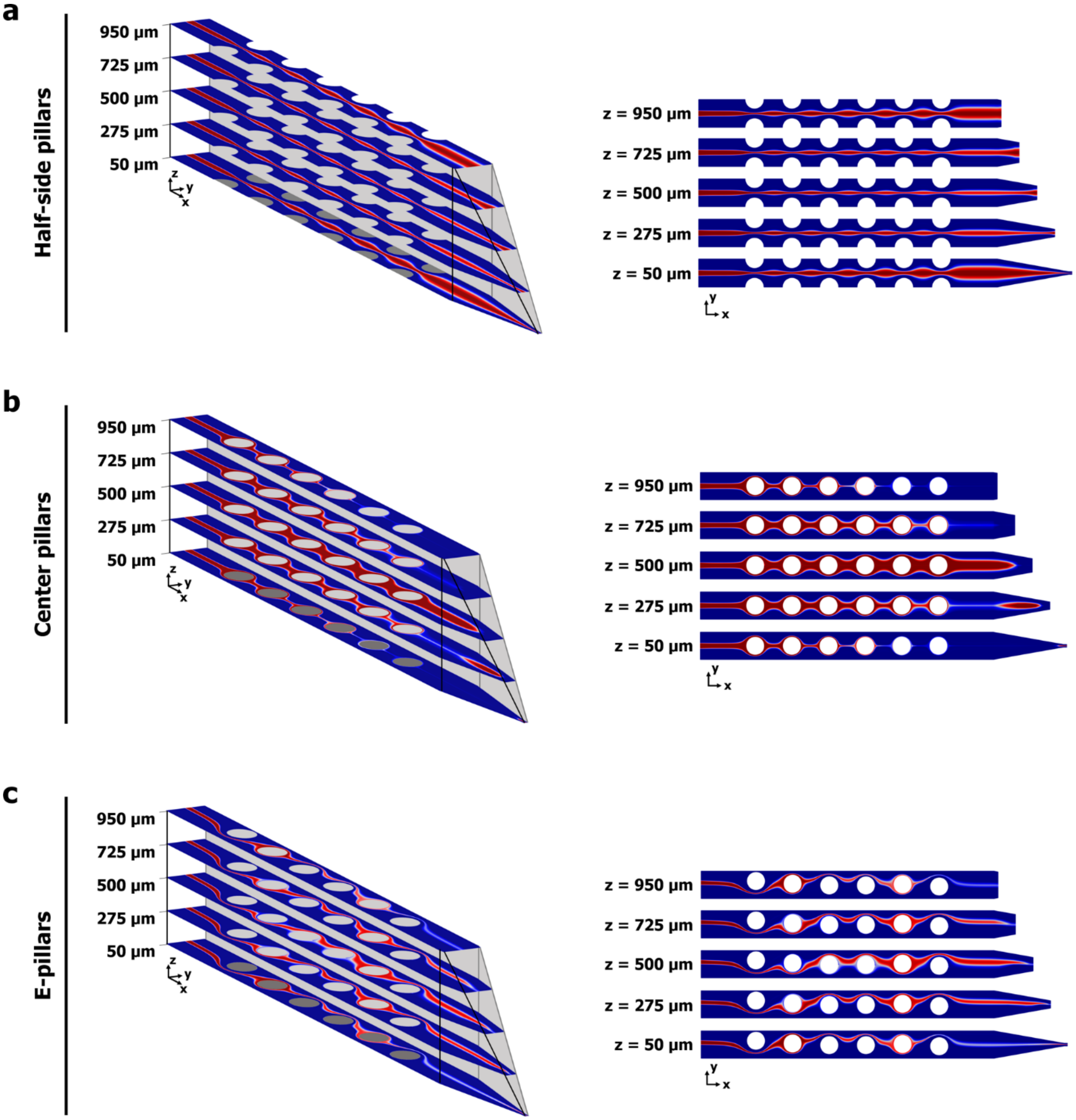

Figure S4. Concentration slices: x–y concentration slices at five z locations for the **a)** half-side pillar channel, **b)** center-pillar channel, and **c)** E-channel.

[Figure S5]

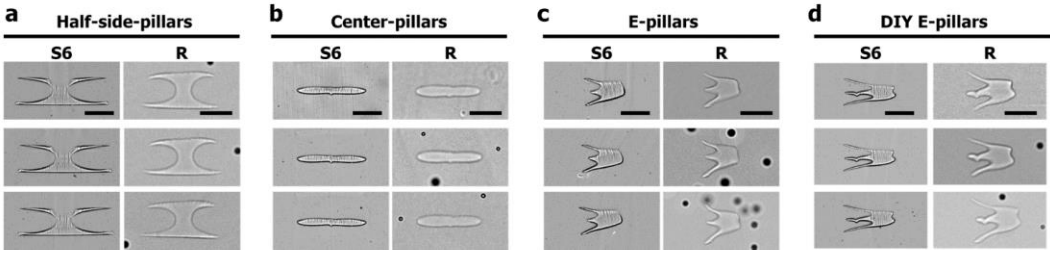

Figure S5. Cross-sectional particle optical microscope images. Additional particles are shown at location S6 and R for **a)** half-side pillar, **b)** center-pillar, **c)** E-pillar, and **d)** DIY E-pillar channels. Scale bars for columns S6 represent 1 mm. Scale bars for columns R represents 100 µm.

[Figure S6]

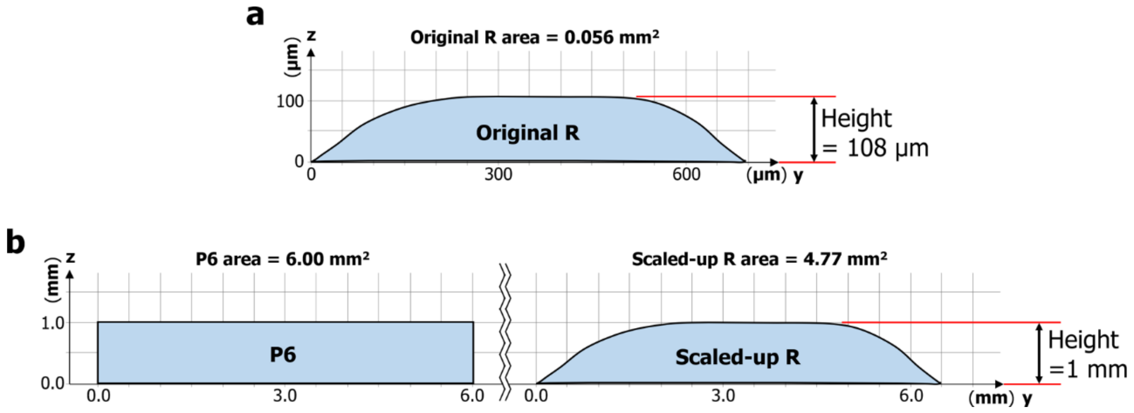

Figure S6. Scaling of the reduction channel cross-sectional area. a) The original reduction cross-sectional area. **b)** To compare the P6 and R cross-sectional areas, the R cross-section was scaled up by 1000/108 = 9.26 for comparison using consistent units.

[Figure S7]

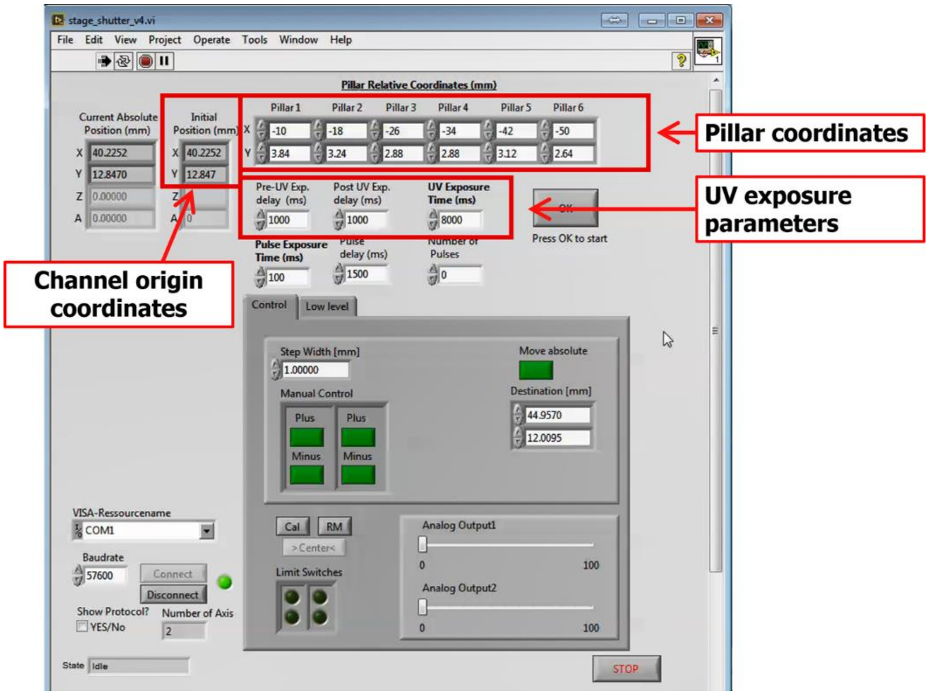

Figure S7. LabVIEW DIY pillar control: Using the custom LabVIEW, the pillar x–y coordinates are input, the channel origin is manually determined, and the UV conditions are set to the desired exposure time.

[Figure S8]

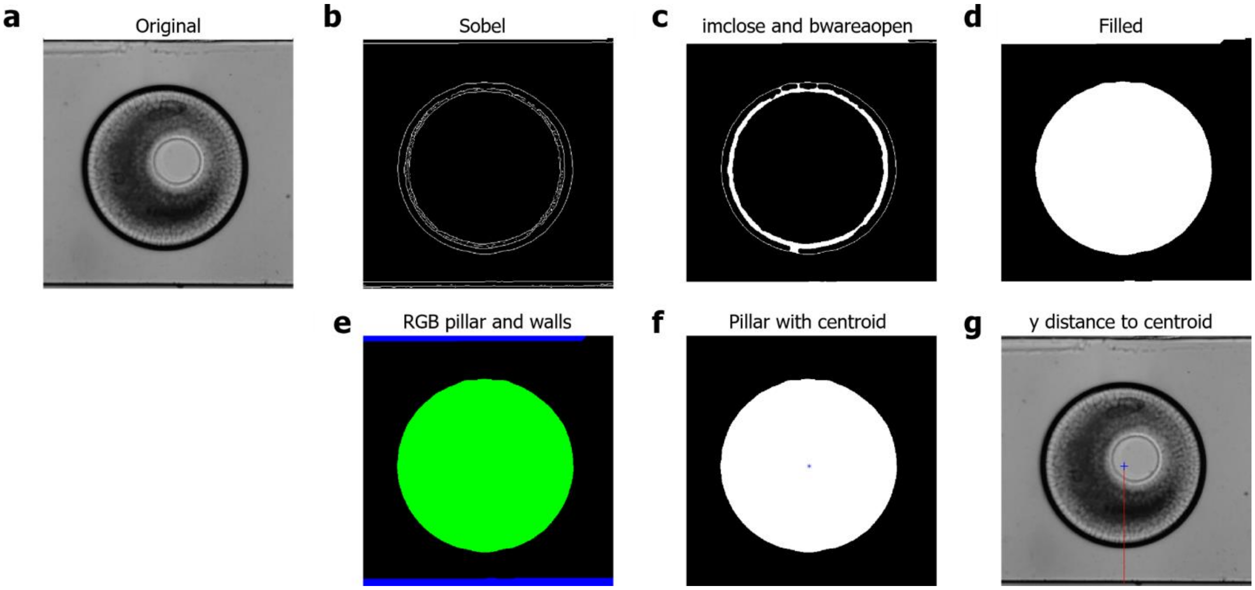

Figure S8. **Measuring pillar properties with MATLAB**. **a)** An image of a pillar is manually cropped, and the Sobel edge detection method **b)** is used to identify the pillar and the channel walls. **c)** Morphological closing and opening operations connect outlines and remove noise. **d)** the pillar and channel walls are filled white. **e)** Pillars are colored in green, while channel walls are colored in blue**. f)** The pillar centroid is identified, and **g)** the distance from the centroid to the channel wall is automatically calculated.

[Figure S9]

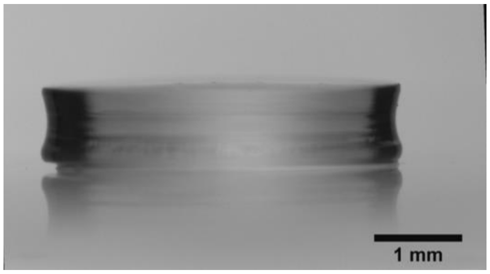

Figure S9. **DIY pillar side profile**. The shape of the DIY pillars is shown from a stereoscopic image of a pillar imaged outside of the channel. The side walls appear convex owing to the 0.085 NA of the 2.5× objective used to for polymerization and scattering of UV light.

[Figure S10]

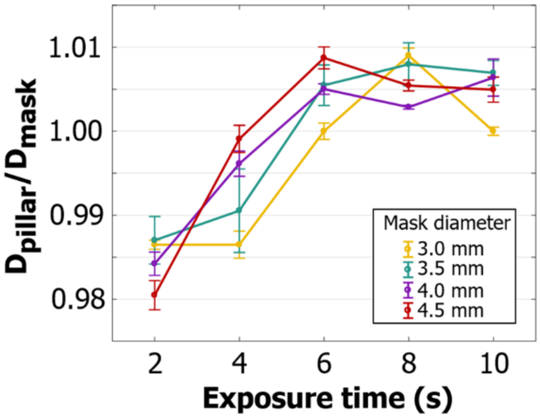

Figure S10. Normalized DIY pillar diameter. The normalized diameters of DIY pillars were measured as a function of UV exposure time using photomasks with four different sizes.

[Supplemental References]

[1] Books with editor: H. Bruus, in *Theoretical Microfluidics*, Ch. 4, 71–90 Oxford University Press, NY, USA **2007**, pp. 71–90.
[2] M. Kim, Y. Huang, K. Choi, C. H. Hidrovo, *Microelectron. Eng*. **2014**, *124*, 66.
